# Medical management of post-sublobar resection pulmonary granulomatous lesion: a report of two cases

**DOI:** 10.1186/s40792-024-01969-9

**Published:** 2024-08-21

**Authors:** Hideki Endoh, Nariaki Oura, Satoru Yanagisawa, Nobutoshi Morozumi, Nobuhiro Nishizawa, Ryohei Yamamoto, Yukitoshi Satoh

**Affiliations:** 1 Department of Thoracic Surgery, Saku Central Hospital Advanced Care Center, 3400-28 Nakagomi, Saku, Nagano, 385-0051 Japan; 2 Department of Respiratory Medicine, Saku Central Hospital Advanced Care Center, 3400-28 Nakagomi, Saku, Nagano, 385-0051 Japan; 3 Department of Thoracic Surgery, Kitasato University School of Medicine, 1-15-1 Kitasato, Minami-Ku, Sagamihara, Kanagawa 252-0374 Japan

**Keywords:** Corticosteroids, Inflammation, Lung cancer, Mycobacterium, Pulmonary segmentectomy, Staple granuloma

## Abstract

**Background:**

Automatic stapling devices are commonly utilized in pulmonary resections, including sublobar segmentectomy. Large tumors can develop around the staple line, posing challenges in distinguishing them from cancer recurrence or inflammatory changes. In this report, we present two cases of symptomatic staple granulomatous lesion effectively managed with medications.

**Case presentation:**

A 74-year-old man presented with a persistent cough and sputum production six years post-segmentectomy for a hamartoma in the left upper lobe. Chest computed tomography (CT) revealed a large tumor around the staple line. Laboratory investigations and bronchoscopic examination revealed no malignancy. The patient received corticosteroids and a cyclooxygenase-2 inhibitor; despite experiencing adverse reactions to steroids, both tumor size and respiratory symptoms were significantly reduced. The second case involved a 78-year-old woman who underwent pulmonary resection for suspected lung cancer. Despite a non-malignant tumor diagnosis, she reported a cough six months post-surgery. Chest CT revealed extensive shadow around the surgical staple, which was diagnosed as mycobacterium granuloma. Low-dose erythromycin induced inflammatory changes but effectively reduced the lesion.

**Conclusions:**

Granulomatous lesions around the staple can be effectively managed with medication, and monitoring the treatment response proves valuable in distinguishing them from tumor recurrence post-pulmonary resection.

## Background

An automatic stapling device plays a crucial role in facilitating the surgical resection and closure of lung tissue. However, suture or staple granulomas can develop after pulmonary resection; occasionally, large tumors can also occur around the staple line. Distinguishing granulomas from cancer recurrence or other inflammatory changes poses challenges [1]. Suture granulomas can yield false-positive results on fluorine-18 deoxyglucose-positron emission tomography (FDG-PET) [2–4], and pathological findings are confirmed only after resection.

Herein, we report two cases of symptomatic granulomatous lesions around the staple and their medical management.

## Case presentation

### Case 1

**Fig. 1:**
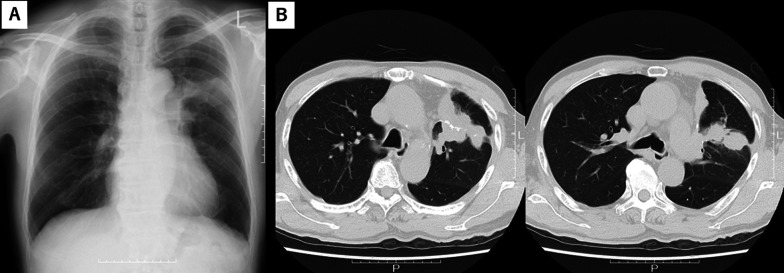
Radiographic findings for case 1. **A** Chest radiography revealed an abnormal shadow in the left upper region. **B** Computed tomography showing a granulomatous tumor of approximately 3 cm in diameter

**Fig. 2:**
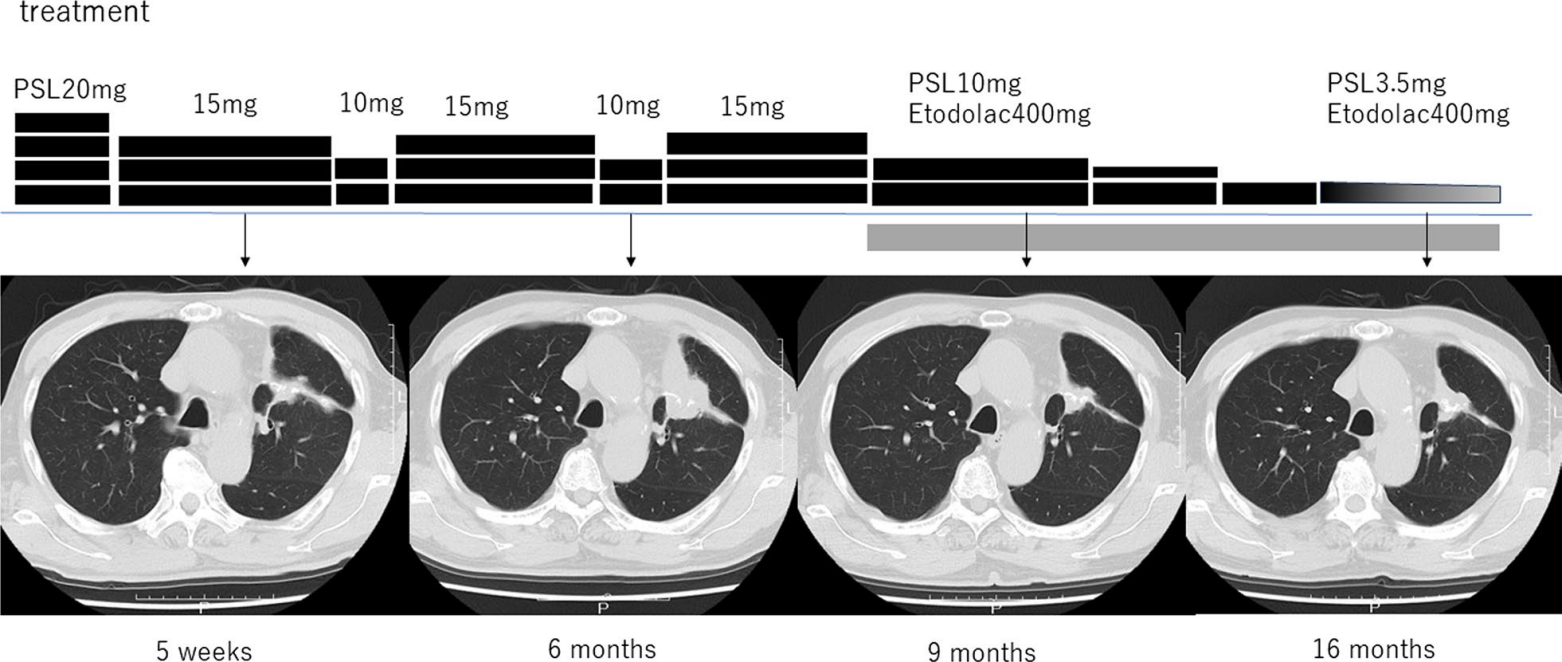
Clinical course and imaging for case 1. The shadow shrank after the daily use of 15 mg of prednisolone (PSL) and regrew on reduction of the PSL dose. Etodolac prescription helped to control the patient’s symptoms and reduced the mass shadow, following which the PSL dose was tapered

A 74-year-old man presented with persistent cough and sputum production 6 years after S3-segmentectomy for a left upper lobe hamartoma. In the previous surgery, pulmonary resection was performed using an automatic stapling device after ligating and cutting the pulmonary artery (A3) and pulmonary vein (V3) using silk thread. He was prescribed medications for chronic atrial fibrillation and hypertension. Chest radiography and computed tomography (CT) revealed a large tumor (59 × 39 mm) around the staple line (Fig. 1). Laboratory data, including white blood cell count, C-reactive protein, and carcinoembryonic antigen, were within normal limits; results for tuberculosis-specific interferon-gamma and beta-D-glucan were negative. Bronchoscopic examination did not reveal malignant tumor cells, mycobacteria, or aspergillus, and the tumor was diagnosed as a *granuloma*. To reduce granulomatous reactions and associated symptoms, a corticosteroid was prescribed alongside an antitussive drug. Prednisolone initiation at 20 mg/day for one week, followed by a sustained dosage of 15 mg/day, led to a notable reduction in cough, sputum, and tumor size a month post-prescription. However, the tumor regrew after reducing the prednisolone dose to 10 mg/day, with this reduction-related failure recurring twice. Prednisolone dosage was increased to 15 mg/day, and after confirming improved shadow, cyclooxygenase-2 inhibitor (etodolac) was recommended, and prednisolone dose was tapered. The patient experienced steroid-related side effects, including cataracts and muscular pain, with myopathy requiring crutch walking. Finally, the medication was discontinued after the chest image revealed a complete resolution of the abnormal shadow (Fig. 2). He has been healthy with no recurrence for 4.5 years and is medication free.

### Case 2

A 78-year-old woman, initially referred for suspected lung cancer, presented with a 1-cm-diameter nodule at the center of the right S6 segment. As the tumor was located near a branch of the pulmonary artery (A6a), A6a was ligated and cut by silk thread and a partial resection was performed by inserting an automatic stapling device into the lung parenchyma to allow a rapid pathological examination. Following partial resection of the right lung, the tumor was diagnosed as a granulomatous lesion without malignancy. Laboratory examinations revealed no infectious causes.

**Fig. 3:**
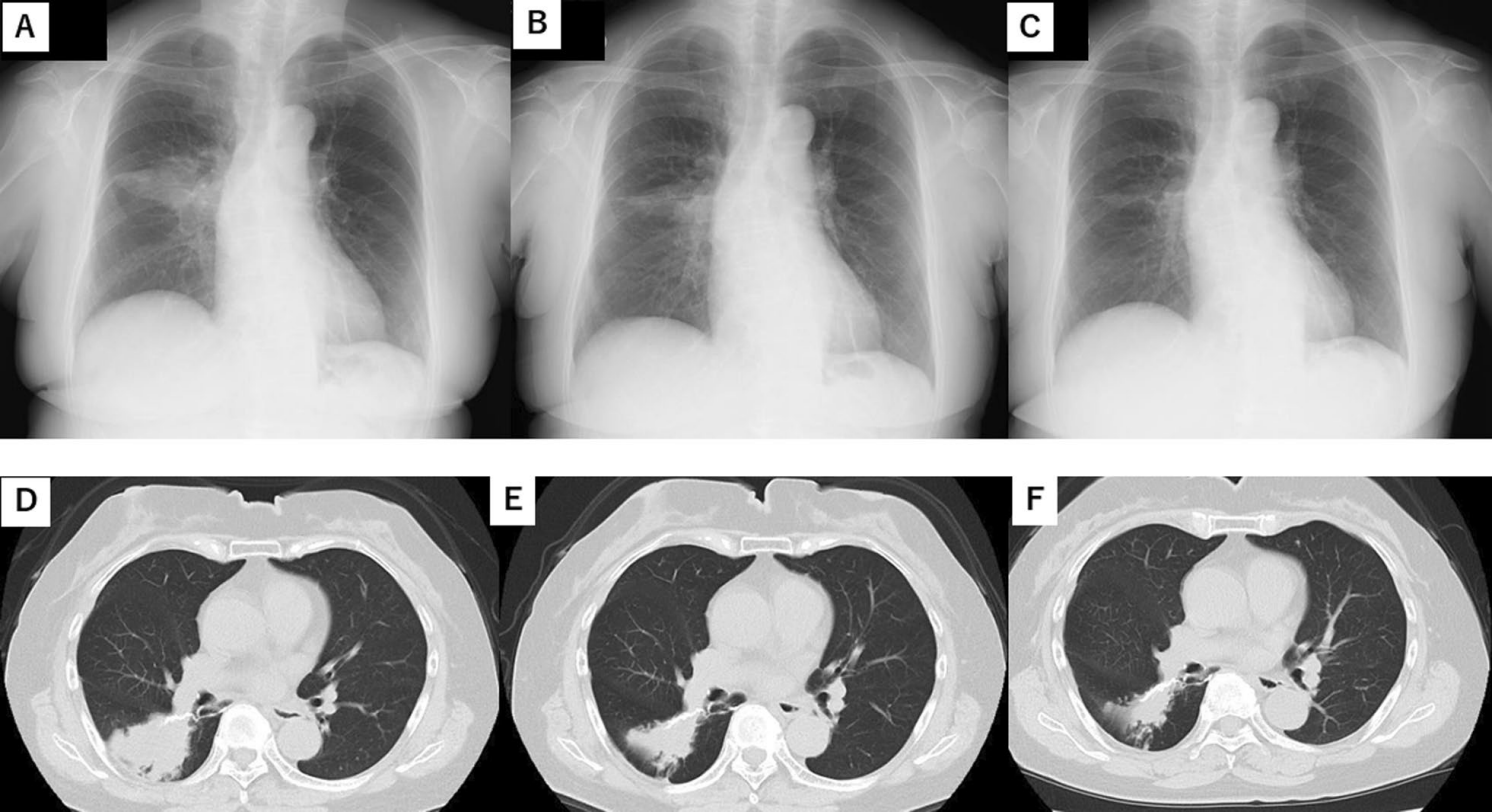
Radiographic findings for case 2. **A**, **D** Chest radiograph and computed tomography (CT) image six months after partial resection of the right lower lobe shows the shadow of a suspected mass. **B**, **E** Imaging shows that the shadow shrunk four months after the use of low-dose erythromycin. **C**, **F** Chest radiograph and CT image show well-controlled inflammatory change after one year

Six months post-resection, the chest CT revealed a granulomatous lesion measuring > 4 cm in diameter around the staple-line. Laboratory data indicated a positive Mycobacterium avium complex (MAC) antibody with no potential malignancy. In response, the physician prescribed low-dose erythromycin (400 mg/day). Two months later, the shadow and cough were significantly reduced, and erythromycin was continued without any noticeable adverse events (Fig. 3). She has continued medications and follow-up without any adverse events for 2.5 years after starting erythromycin.

## Discussion

Recently, sublobar resections for lung cancer have demonstrated comparable effects to lobar resections in terms of patient survival [5]; however, vigilance is required for careful observation of local recurrence around the residual lobe. In some cases, surgical resection has been followed by the development of suture granulomas [6–8].

The detection of lung cancer recurrence, both locally and surrounding the staple line, typically involves identifying a tumor on CT, followed by FDG-PET [1]. However, PET findings can be perplexing as both granulomas and cancers accumulate FDG. False-positive reports of suture granulomas on FDG-PET at the surgical margin have been documented in the lungs, stomach, colon, ovary, and urological neoplasm [2–4, 9]. Okazaki et al. reported similar findings of granuloma formation after segmentectomy by electrocautery caused by a polyglycolic acid sheet but not a staple [10]. For the present cases, FDG-PET could not be performed because the previous pathological diagnosis was not cancer. Had cancer recurrence been suspected, the decision or FDG-PET and clinical course and/or re-examination of therapy would be needed; however, an accurate diagnosis by imaging study is difficult.

Usuda et al. reported the utility of diffusion-weighted magnetic resonance imaging in differentiating between suture recurrence and granulomas [2]. Matsuura et al. emphasized the difficulty in distinguishing between staple-line granulomas and recurrence after sublobar resection for lung cancer, correlating staple line recurrence with vascular invasion, surgical margin, nodular thickening, and a tendency for thickening to progress [11, 12].

In the first case, considering the tumor diagnosis owing to a previous surgery performed for a benign tumor, cytological evaluation of bronchial tissue was performed as an additional examination. While routine observation is standard after diagnosis, this case required medical treatment. Granulomatous lesions may be related to an immunological reaction, and some reports have described well-controlled cases with steroids, infliximab, and cyclooxygenase-2 (COX-2) inhibitors [13–15]. COX-2 inhibitors are proinflammatory during the early stage of inflammation but may also resolve inflammation during later stages [16]. COX-2 inhibitors reduce the production of proinflammatory prostaglandins and have potent anti-neoplastic activity.

In the second case, the suspected tumor mass could have been an infectious nodule associated with non-tuberculous mycobacterium. However, preoperative laboratory tests were negative for anti-MAC antibodies, and other findings of infectious diseases in the resected tissue were undetected. A partial resection was performed by the insertion of an automatic stapling device into the lung parenchyma after cutting at A6a. Besides resection for the malignant tumor, wedge resection should be performed widely to ensure adequate margin. The observed postoperative mass could be attributed to residual non-tuberculous mycobacterium infection. Occasionally, granulomatous lung lesions are caused by non-tuberculous mycobacteria [17], and serum anti-MAC antibodies are considered a substitute for bronchoscopic examinations. Yoshino et al. reported a case of complete lobectomy after segmentectomy, resulting in a granuloma attributed to MAC [13]. In their case, the mass shrank in size with steroids; however, after 39 months, regrowth of a massive shadow occurred, and the re-administering of steroids had no effect. A retrospective study reported that long-term low-dose erythromycin monotherapy may suppress the exacerbation of MAC lung disease, while ensuring well-tolerated adverse events [18, 19]. Macrolides, such as erythromycin, promote macrophage-led phagocytosis of apoptotic cells, preventing further inflammation [20]. Although the exact mechanism by which macrolides control the granulomatous reaction is unclear, the resolution of the granuloma was observed with erythromycin monotherapy in our second case.

## Conclusions

We reported two cases of staple granulomas following sublobar resection of a benign lung tumor. These cases demonstrated the resolution of granulomatous lesion with the administration of prednisolone, COX-2 inhibitors, or antibiotics. In one case, strong adverse events prompted a reduction in steroid dosage, leading to the prescription of alternative medication. Therefore, vigilant assessment for tumor recurrence after surgical resection is crucial, and the use of medications can be helpful in the diagnosis and management of granulomatous reactions.

## Data Availability

Data sharing is not applicable to this article as no datasets were generated or analyzed during the current study.

## Abbreviations

FDG-PET: 18-Fluoro-2-deoxy-glucose positron emission tomography
MAC: Mycobacterium avium complex
COX-2: Cyclooxygenase-2
CT: Computed tomography
